# Supplementary material for: Proanthocyanidin-enriched cranberry extract induces resilient bacterial community dynamics in a gnotobiotic mouse model
Source: Microb Cell. 2021 Apr 29;8(6):131–42. doi: 10.15698/mic2021.06.752 (PMC8144911; doi:10.15698/mic2021.06.752)
Supplement: Supplementary file 1 [file mic-08-131-s01.pdf]

Proanthocyanidin-enriched cranberry extract induces resilient bacterial community dynamics in a gnotobiotic mouse model

Catherine C. Neto[1,2,*,**], Benedikt M. Mortzfeld[3,*], John R. Turbitt[1,2], Shakti K. Bhattarai[3], Vladimir Yeliseyev[4], Nicholas DiBenedetto[4], Lynn Bry[4], Vanni Bucci[2,3,**]

[1]Department of Chemistry and Biochemistry University of Massachusetts-Dartmouth, North Dartmouth, MA

[2]UMass Cranberry Health Research Center, University of Massachusetts-Dartmouth, North Dartmouth, MA

[3]Department of Microbiology and Physiological Systems, University of Massachusetts Medical School, Worcester, MA

[4]Massachusetts Host-Microbiome Center, Department of Pathology, Brigham and Women's Hospital, Harvard Medical School, Boston MA

[*]equally contributing authors

[**]co-corresponding authors

Correspondence should be addressed to:

Vanni Bucci, PhD

vanni.bucci2@umassmed.edu or cneto@umassd.edu

368 Plantation St

Worcester, MA 01605

Phone: 774-455-3854

Running title: Cranberry juice extract microbiome dynamics

Keywords: Cranberry extract, Polyphenols, Proanthocyanidins, Microbiome dynamics, Gnotobiotic mouse model, Microbiome resilience, *Akkermansia muciniphila*

Table S1: Putative identification of ion masses in MALDI-TOF MS spectrum of Figure S1.

m/z	Putative Assignment [M+Cs$^+$]
548.986	Benzoyl-hexoside-pentoside
550.558	Cyanidin-3-arabinoside
565.050	Peonidin-3-arabinoside
580.781	Cyanidin-3-galactoside
596.946	Peonidin-3-galactoside / Quercetin-hexosides
613.021	Myricetin-hexoside
653.066	Unknown
667.079	Unknown
700.964	Quercetin-3-O-(6”-benzoyl)-b-galactoside
709.044	Procyanidin A2
742.965	Quercetin-3-O-(6”-p-coumaroyl)-b-galactoside

Table S2: Putative identification of ion masses in MALDI-TOF MS spectrum in Figure S3.

m/z	Putative Structure [M+Cs$^+$]
996.997	(epi)catechin trimer
1285.050	(epi)catechin tetramer
1489.223	Xyloglucan, Hex_5Pent_4
1813.307	Xyloglucan, Hex_7Pent_4
1945.337	Xyloglucan, Hex_7Pent_5
2077.369	Xyloglucan, Hex_7Pent_6
2209.397	Xyloglucan, Hex_7Pent_7
2239.400	Xyloglucan, Hex_8Pent_6
2341.400	Xyloglucan, Hex_7Pent_8
2371.432	Xyloglucan, Hex_8Pent_7
2503.455	Xyloglucan, Hex_8Pent_8
2533.463	Xyloglucan, Hex_9Pent_7

Table S3: Bacterial strains in GnotoComplex 2.0.

Bacterial species	Strain ID
Akkermansia muciniphila	DSM 22959
Anaerostipes hadrus	DSM 3319
Bacteroides cellulosilyticus	DSM 14838
Bacteroides fragilis	ATCC 25285
Bacteroides ovatus	ATCC 8483
Bacteroides vulgatus	ATCC 8482
Bifidobacterium longum subsp. infantis	ATCC 15697
Bilophila wadsworthia	ATCC 51581
Blautia hansenii	DSM 20583
Clostridium hiranonis	DSM 13275
Clostridium ramosum	DSM 1402
Clostridium scindens	ATCC 35704
Coprococcus comes	ATCC 27758
Dorea formicigenerans	ATCC 27755
Eggerthella lenta	DSM 2243
Enterococcus faecalis	ATCC 29200
Escherichia coli	MG1655
Klebsiella oxytoca	ATCC 700324
Lactobacillus reuteri	DSM 20016
Parabacteroides distasonis	ATCC 8503
Prevotella melaninogenica	ATCC 25845
Proteus mirabilis	ATCC 29906
Roseburia hominis	DSM 16839
Ruminococcus obeum	ATCC 29174
Veillonella parvula	ATCC 10790

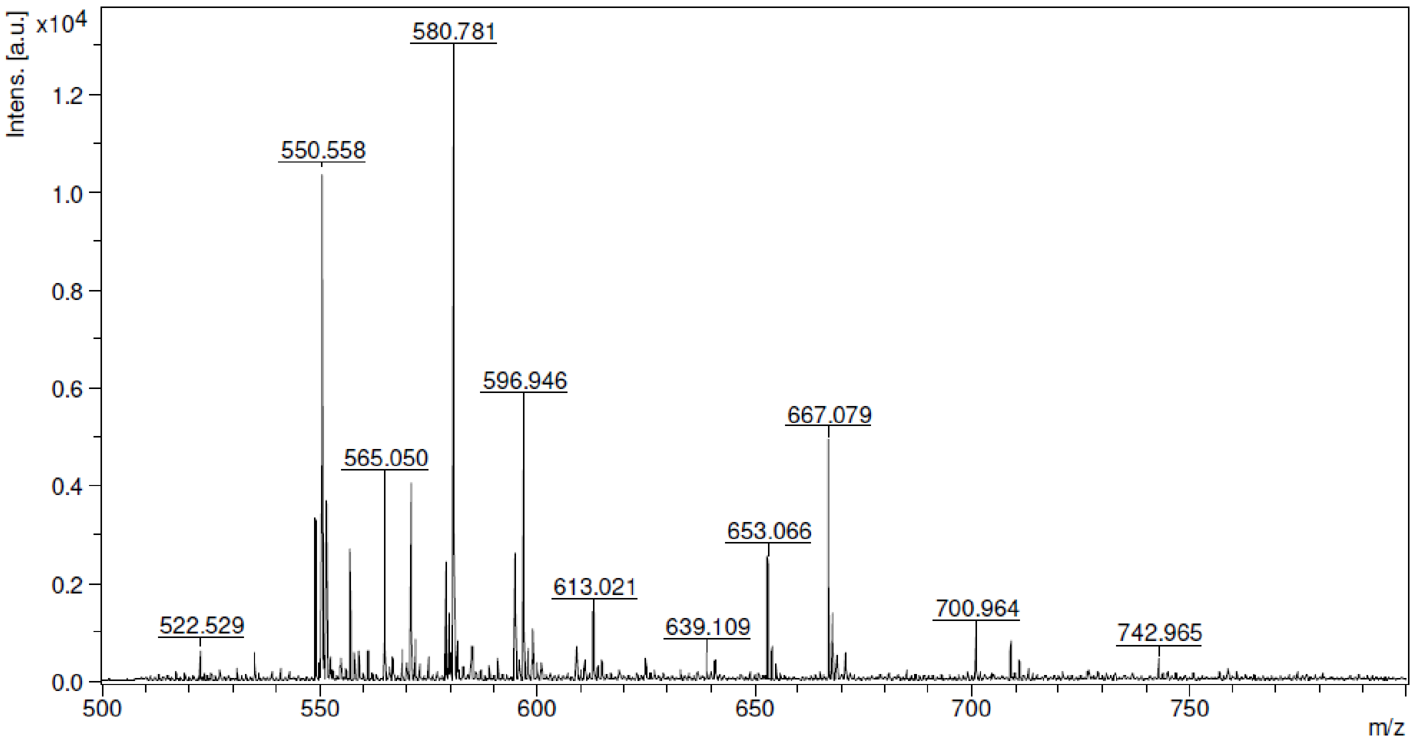

Figure S1: MALDI-TOF MS spectrum of CJE expanded in m/z range 500-800 amu, positive ion mode, CsI_2 added. For putative assignments see Table S1.

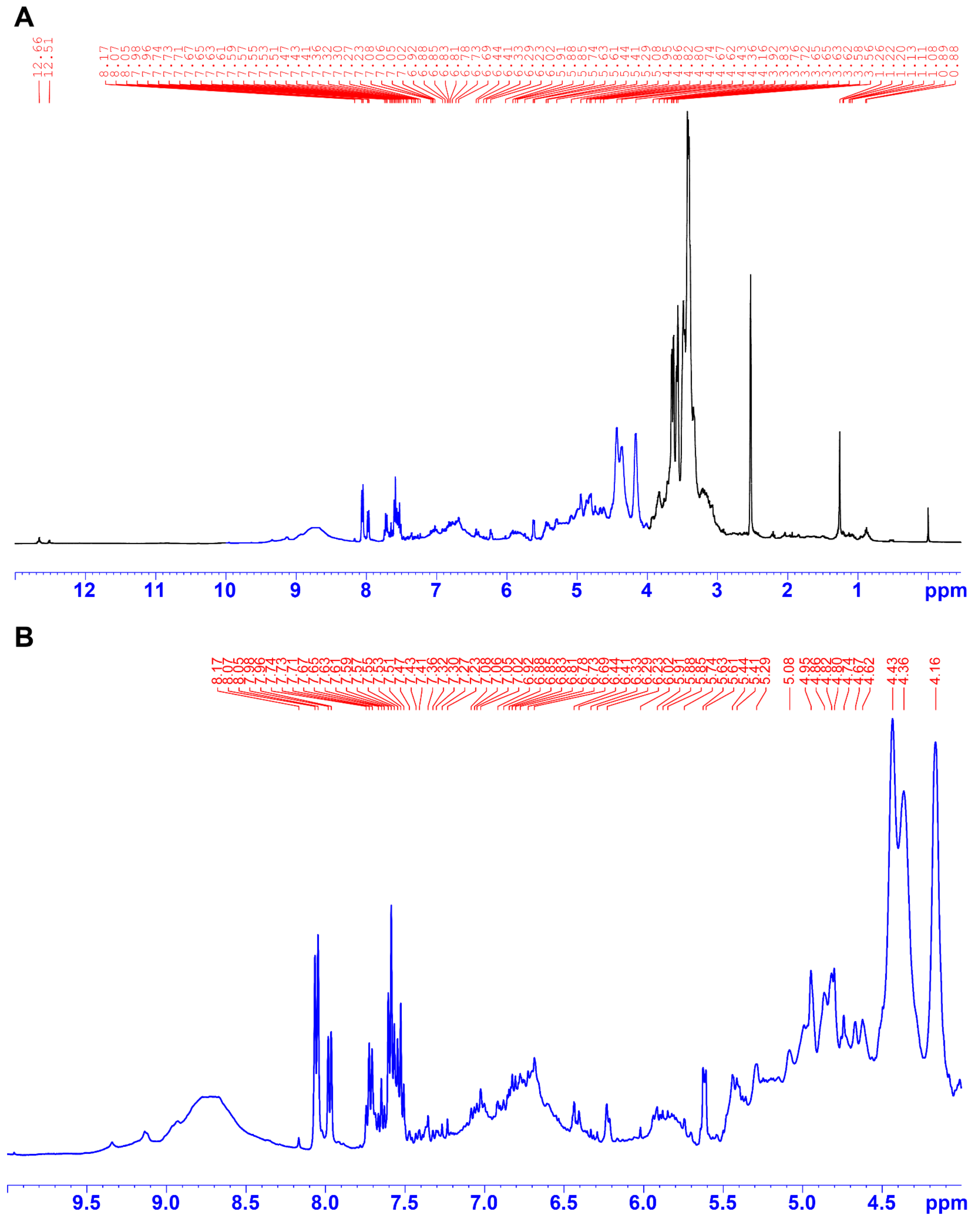

Figure S2: ^{1}H NMR spectrum of CJE in DMSO-d_6 full spectrum (A) and expanded in polyphenol signal region (4-10 ppm) (B).

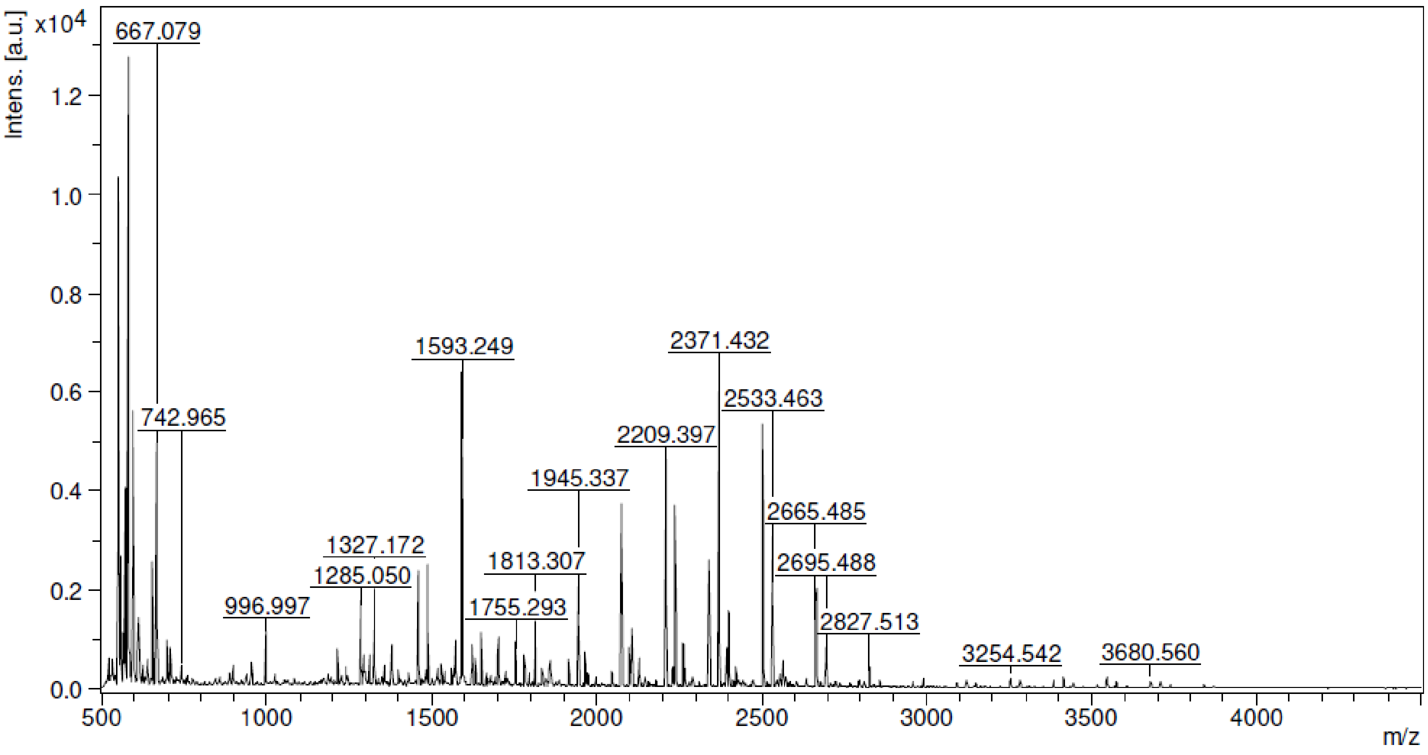

Figure S3: MALDI-TOF MS spectrum of CJE. For putative structures see Table S2. Positive ion mode, CsI_2 added.

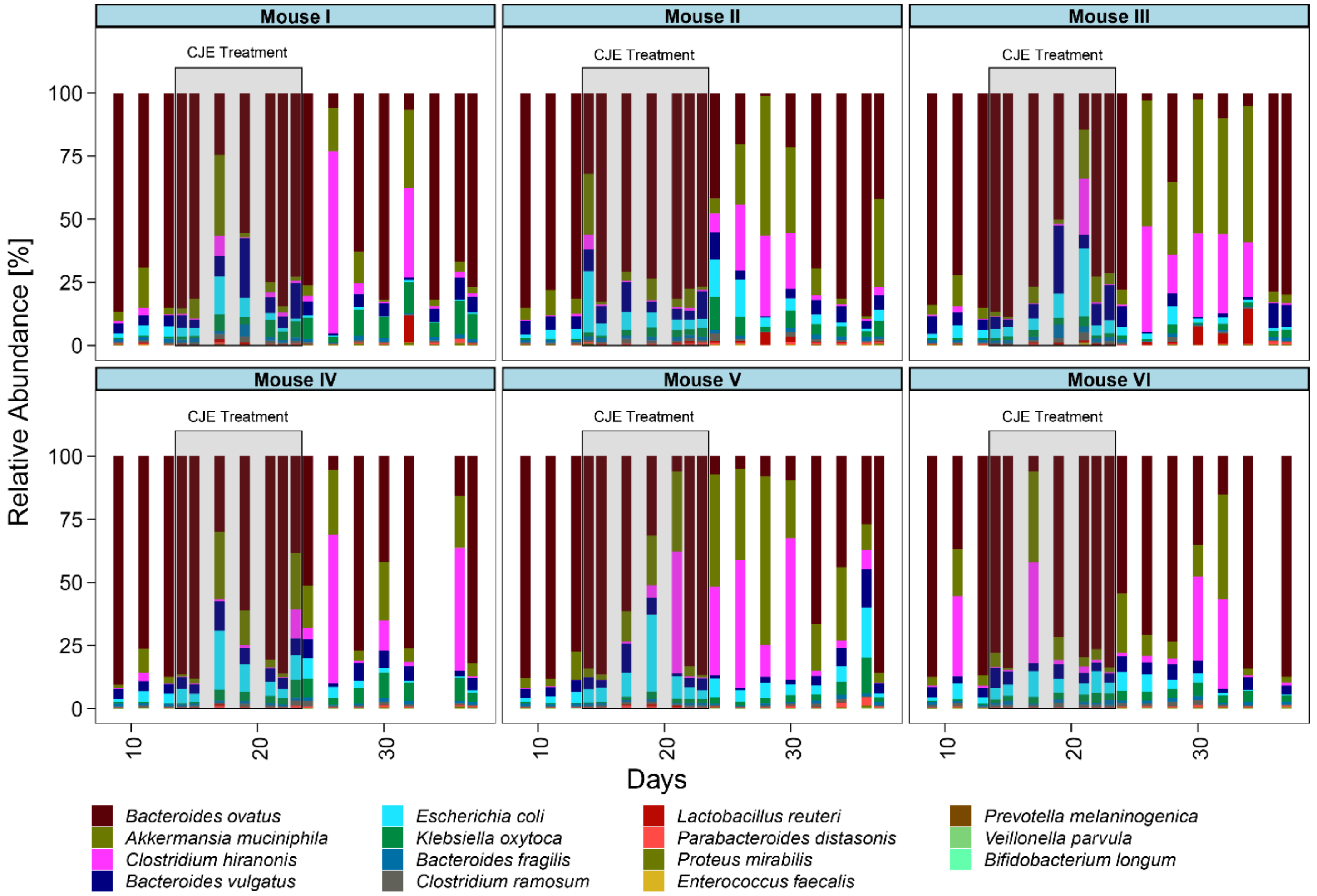

Figure S4: Relative bacterial abundance for the individual mice throughout the cranberry juice extract (CJE) experiment.

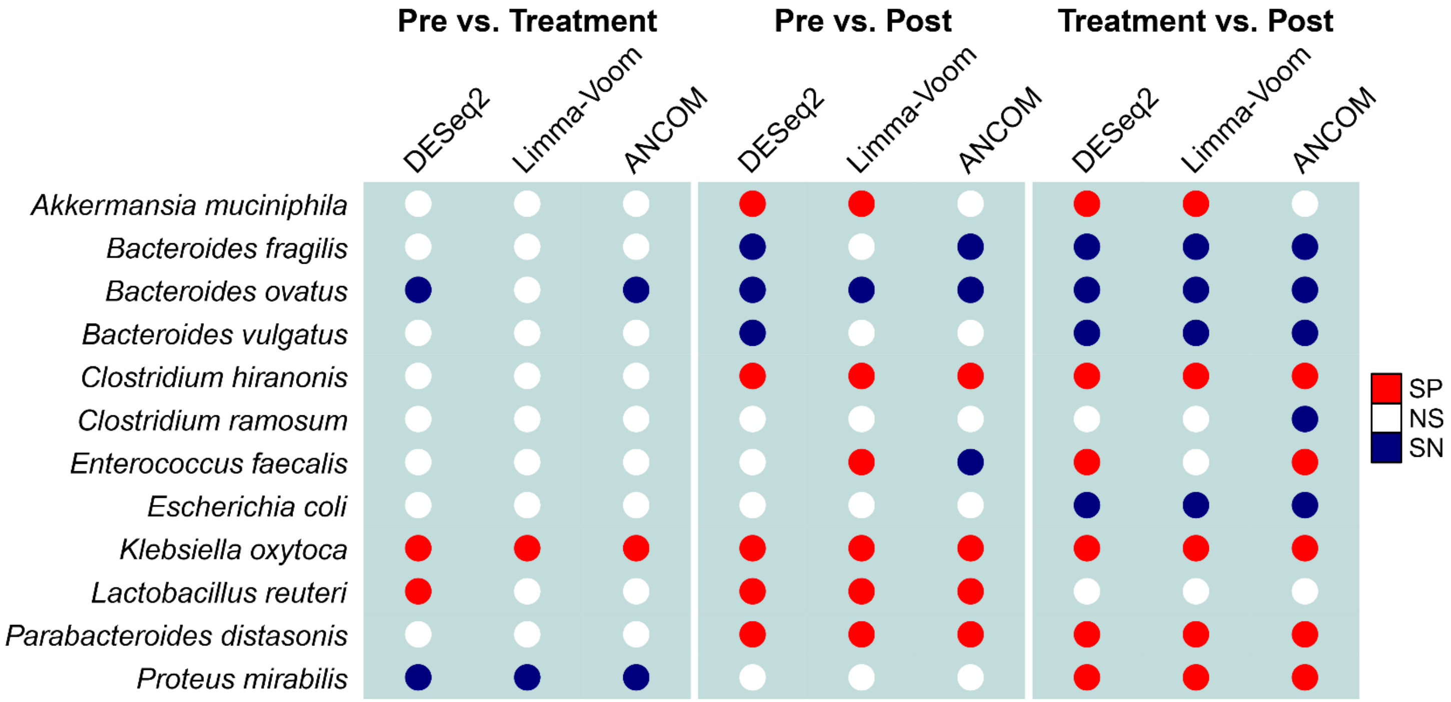

Figure S5: Summary of statistical test outcomes for the three experimental time intervals. Colors indicate no significant change (white, NS), significant positive change (red, SP), and significant negative change (blue, SN) in relative abundance. Significance thresholds are: DESeq $p \leq 0.05$, Limma-Voom $p \leq 0.05$, ANCOM $W > W_{(0.6)}$.

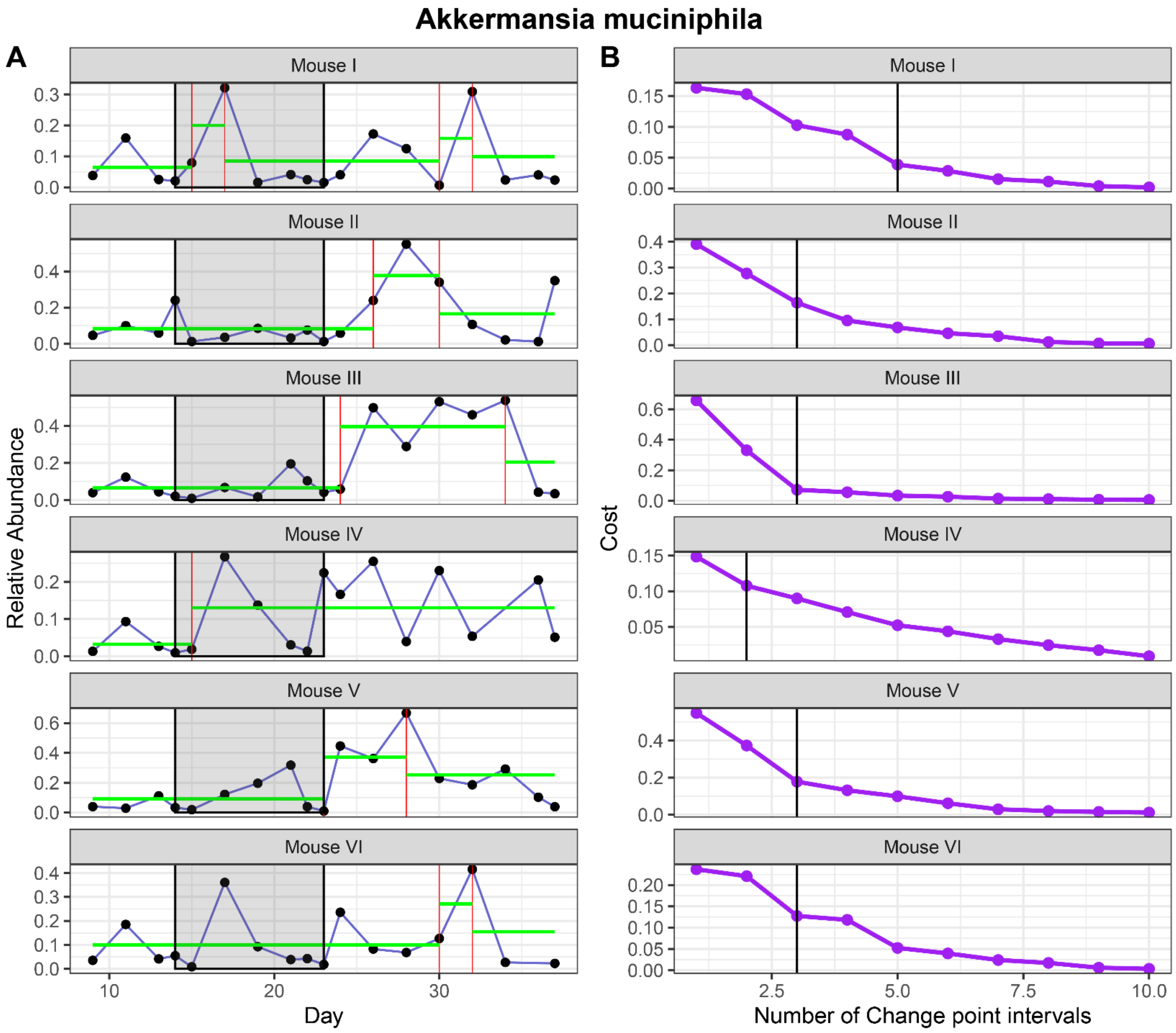

Figure S6: Change point analysis for *Akkermansia muciniphila* (A) and change point interval determination (B).

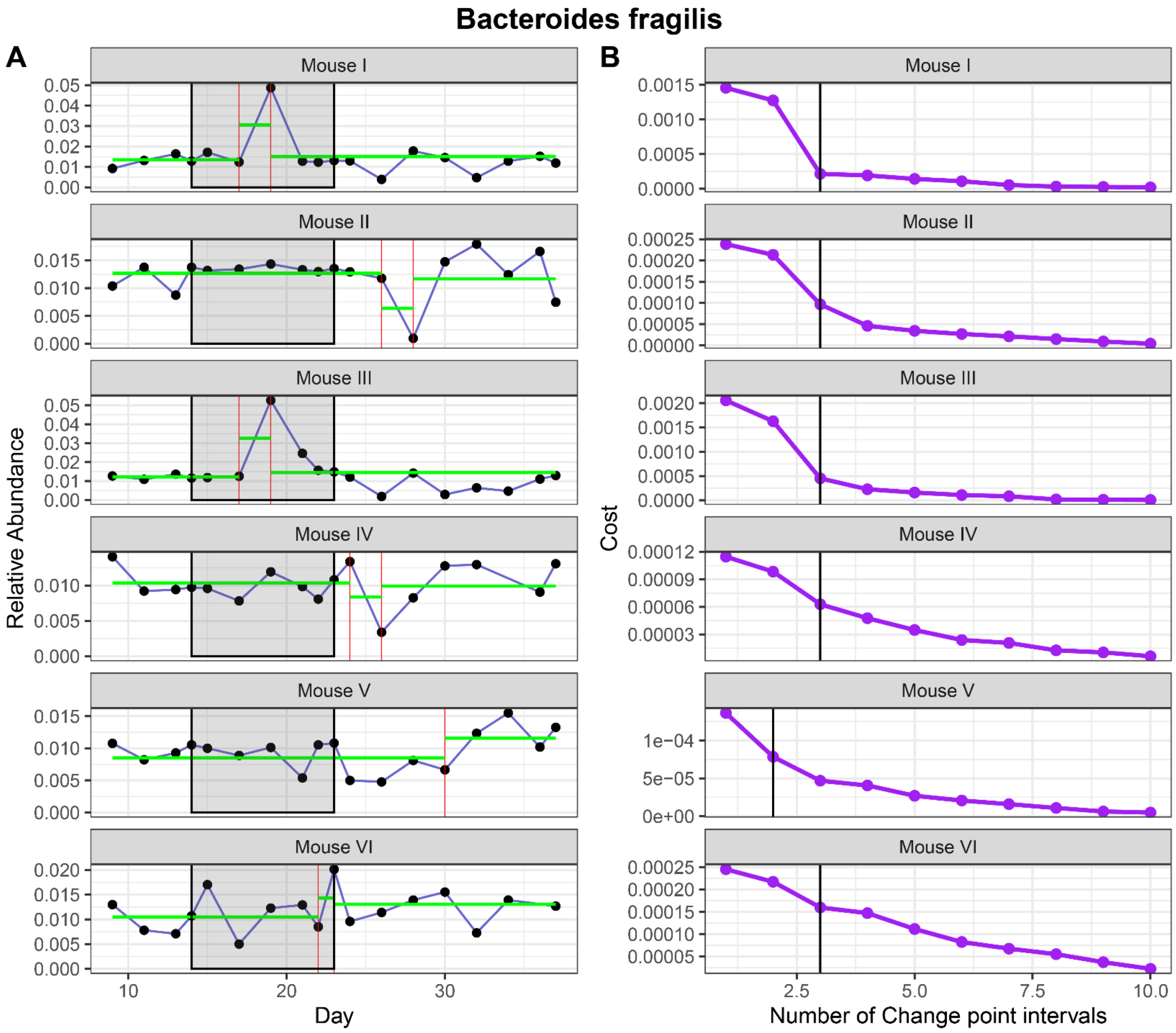

Figure S7: Change point analysis for *Bacteroides fragilis* (A) and change point interval determination (B).

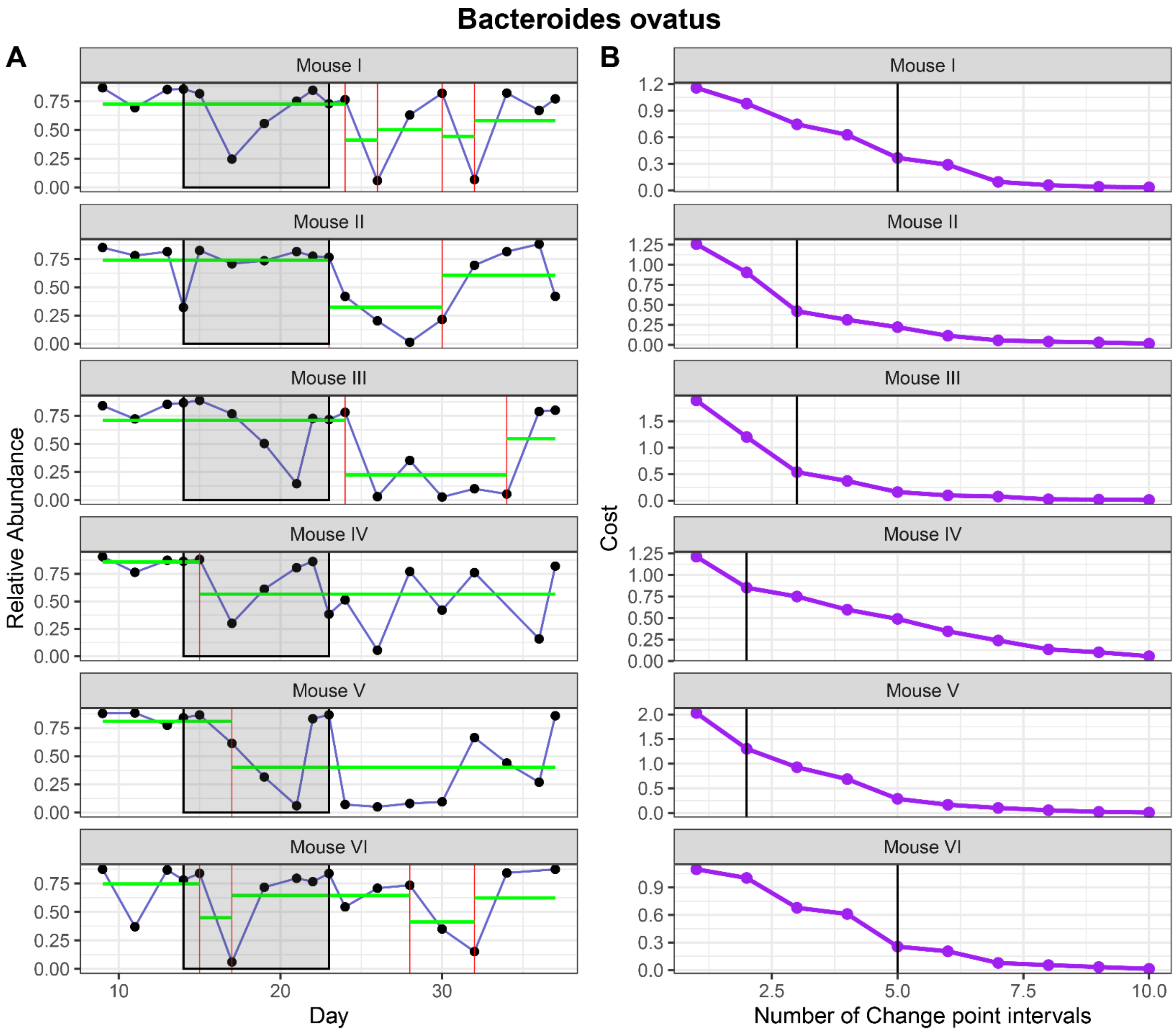

Figure S8: Change point analysis for *Bacteroides ovatus* (A) and change point interval determination (B).

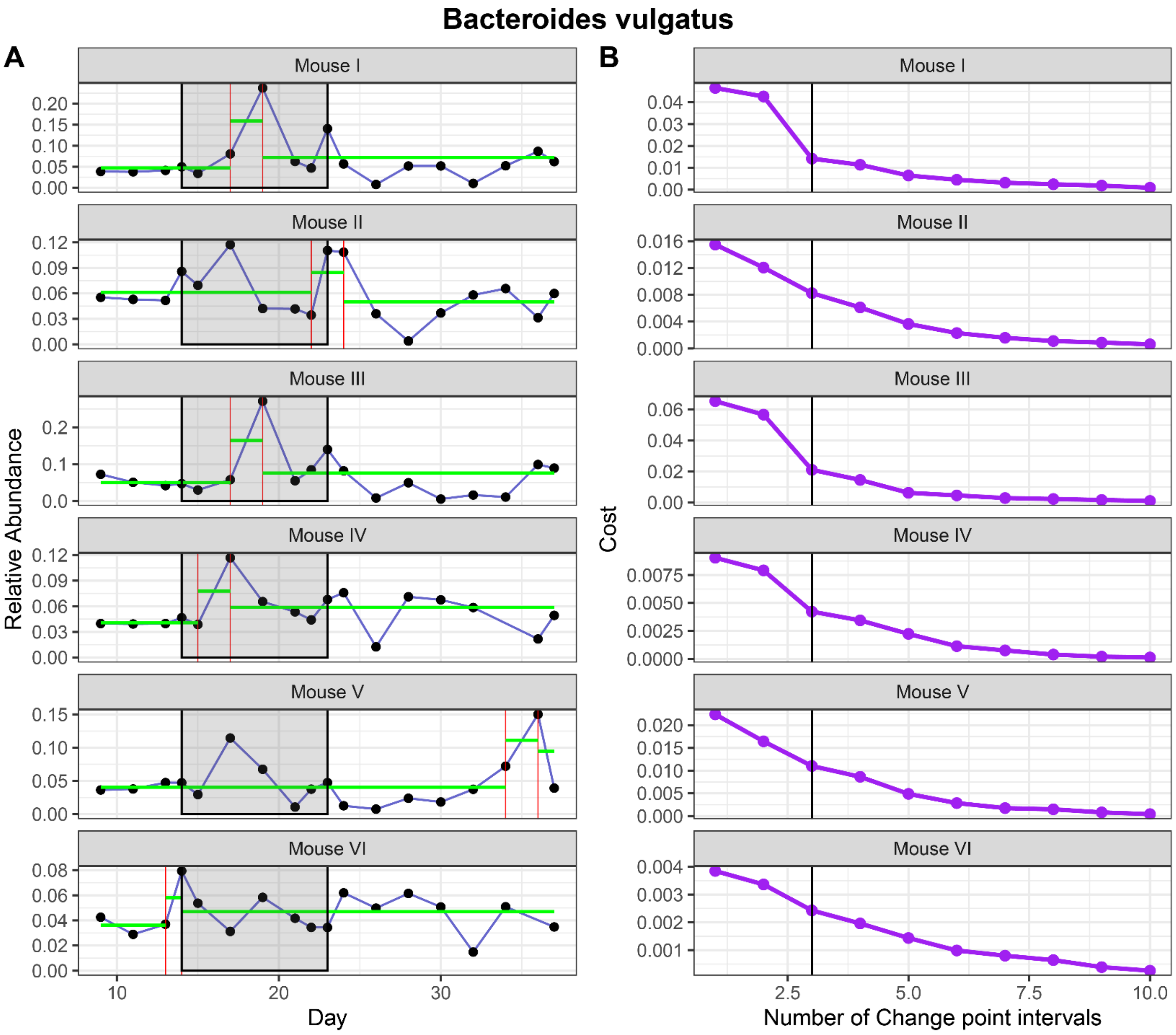

Figure S9: Change point analysis for *Bacteroides vulgatus* (A) and change point interval determination (B).

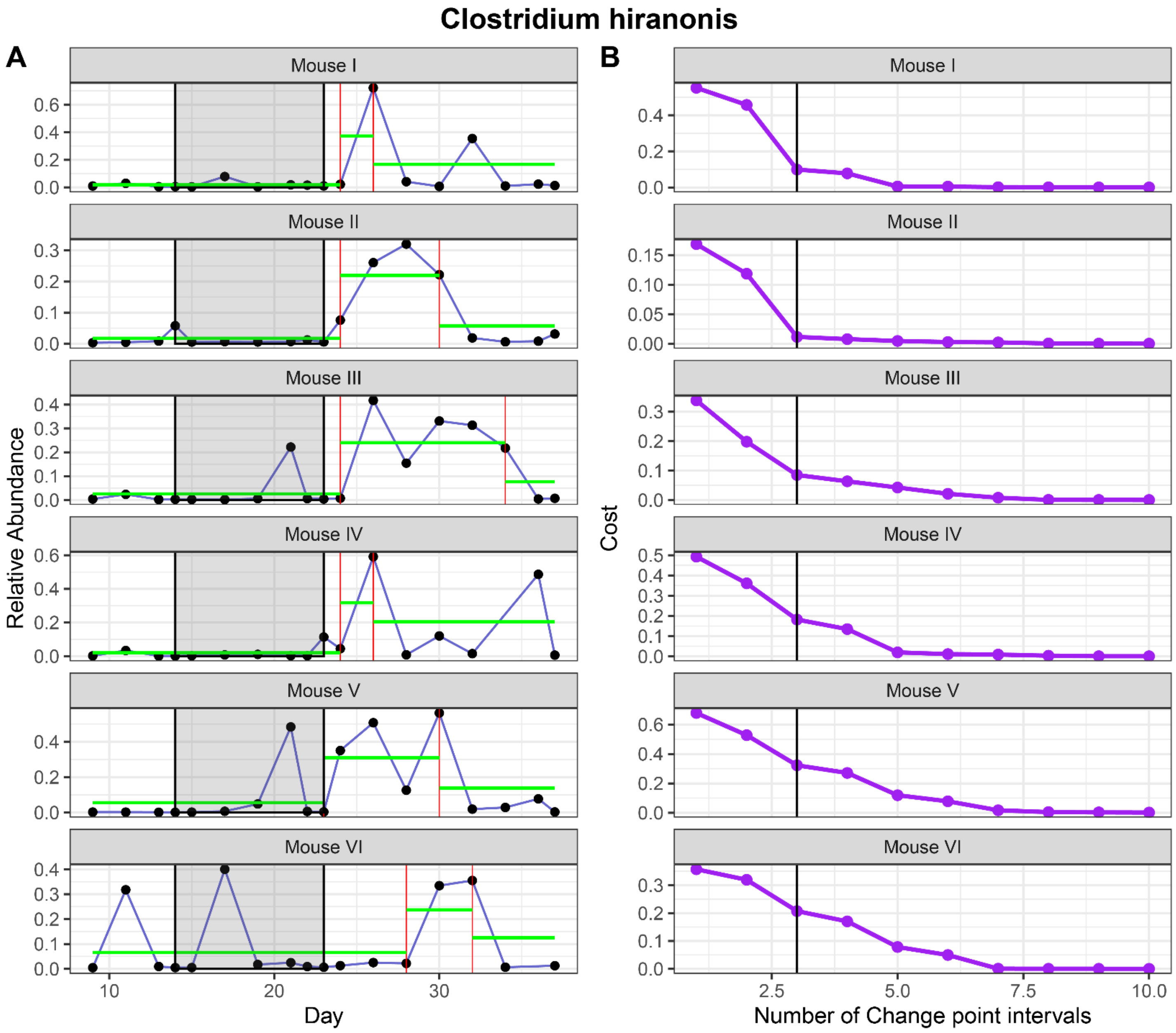

Figure S10: Change point analysis for *Clostridium hiranonis* (A) and change point interval determination (B).

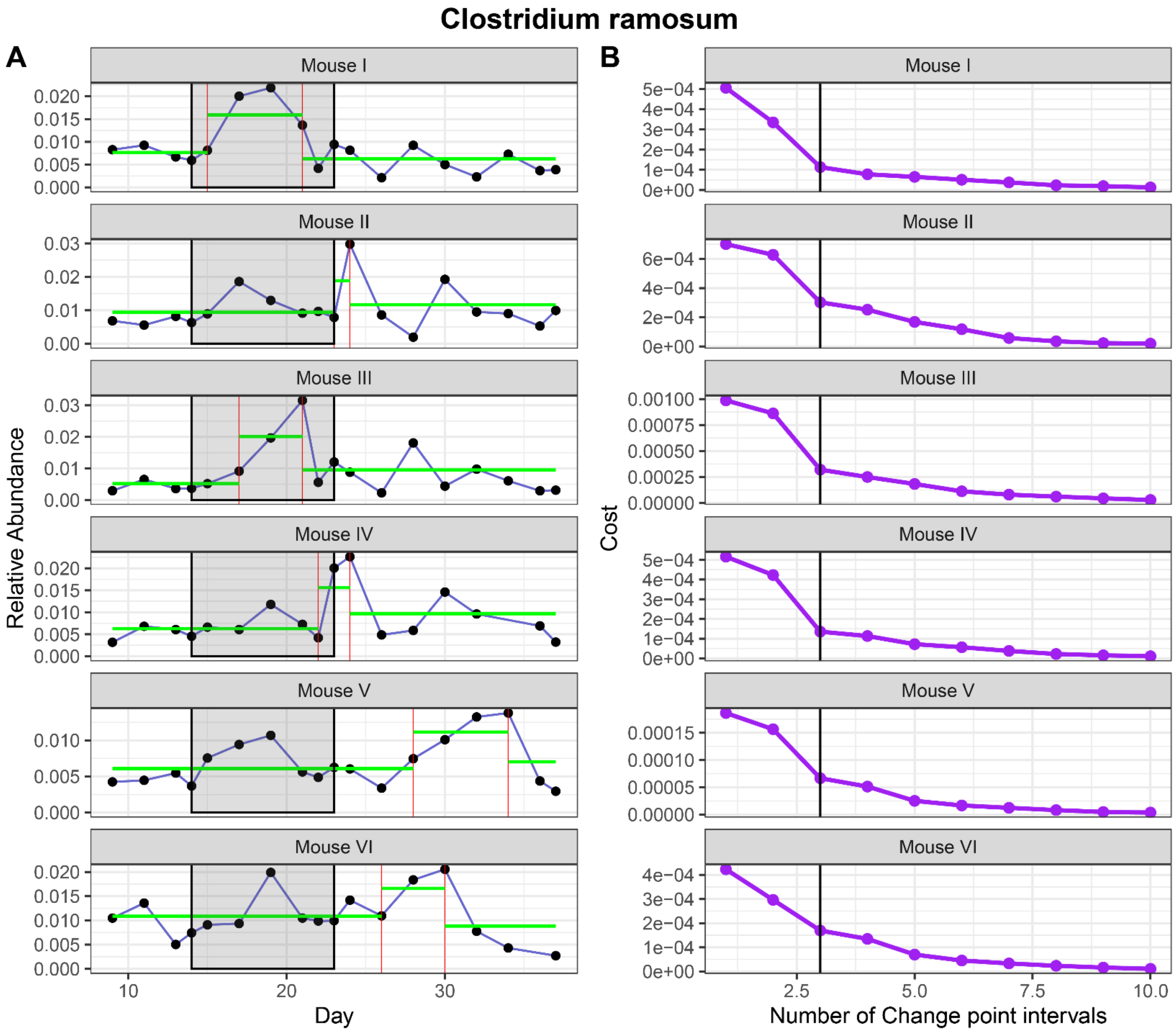

Figure S11: Change point analysis for *Clostridium ramosum* (A) and change point interval determination (B).

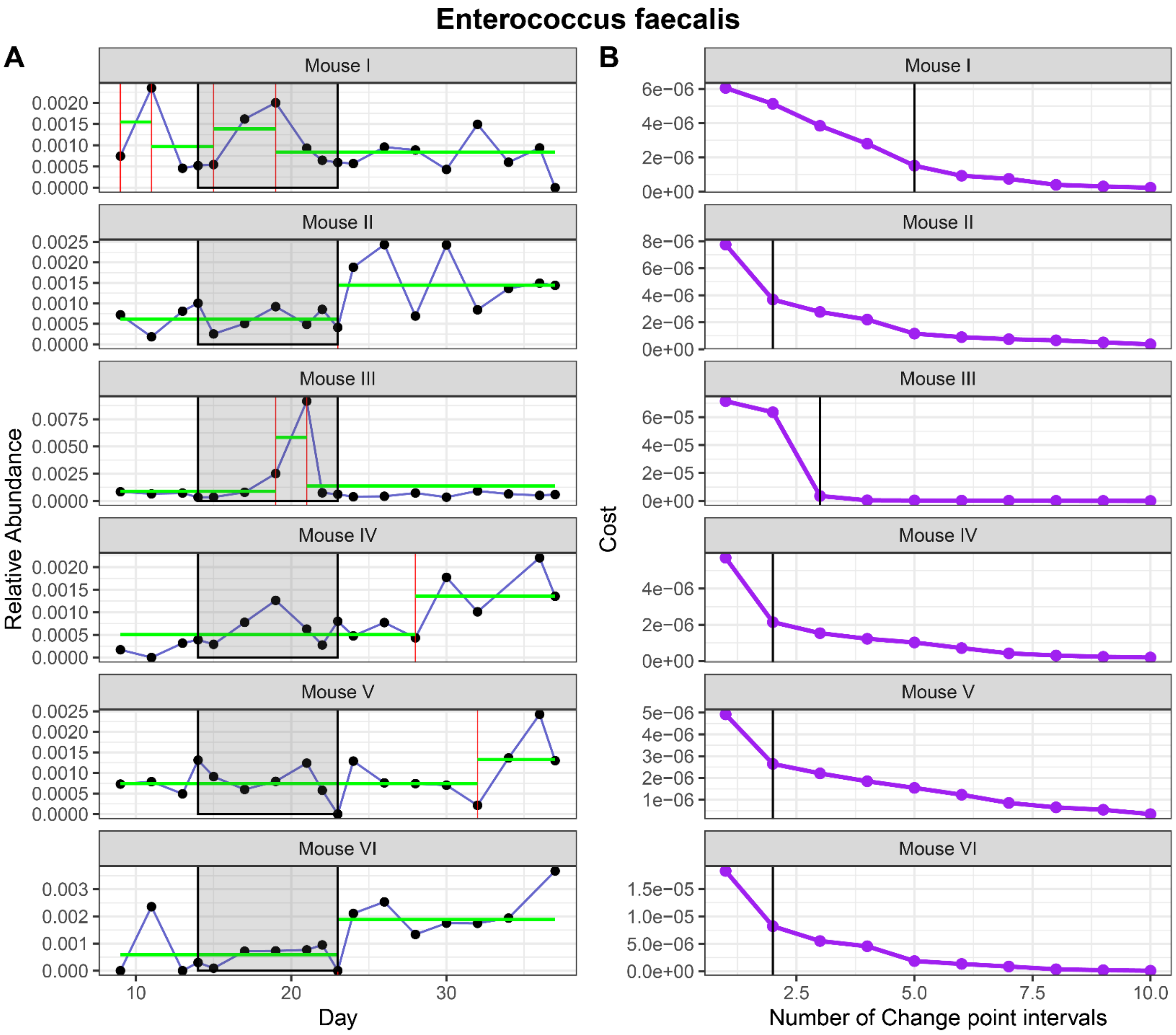

Figure S12: Change point analysis for *Enterococcus faecalis* (A) and change point interval determination (B).

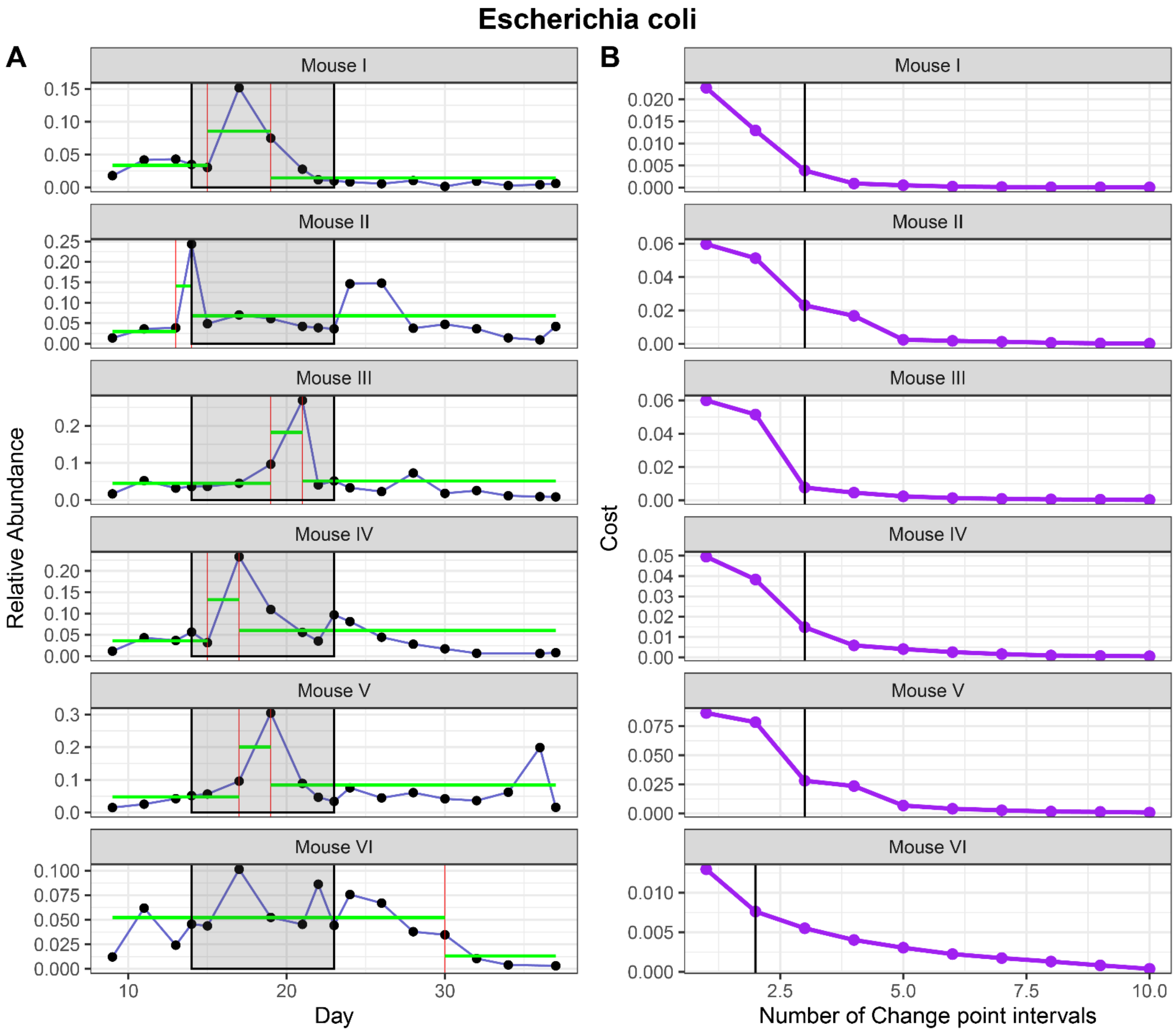

Figure S13: Change point analysis for *Escherichia coli* (A) and change point interval determination (B).

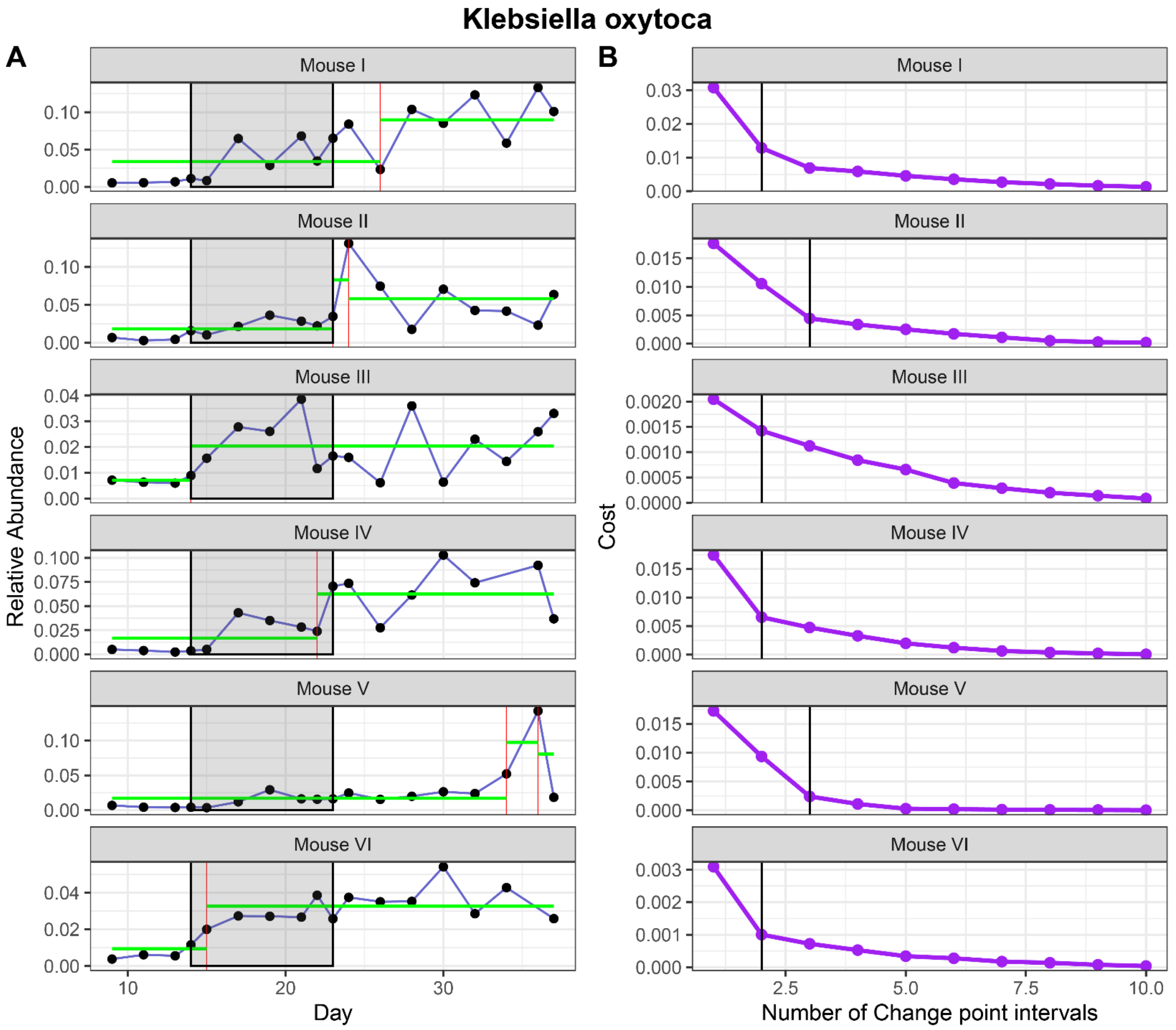

Figure S14: Change point analysis for *Klebsiella oxytoca* (A) and change point interval determination (B).

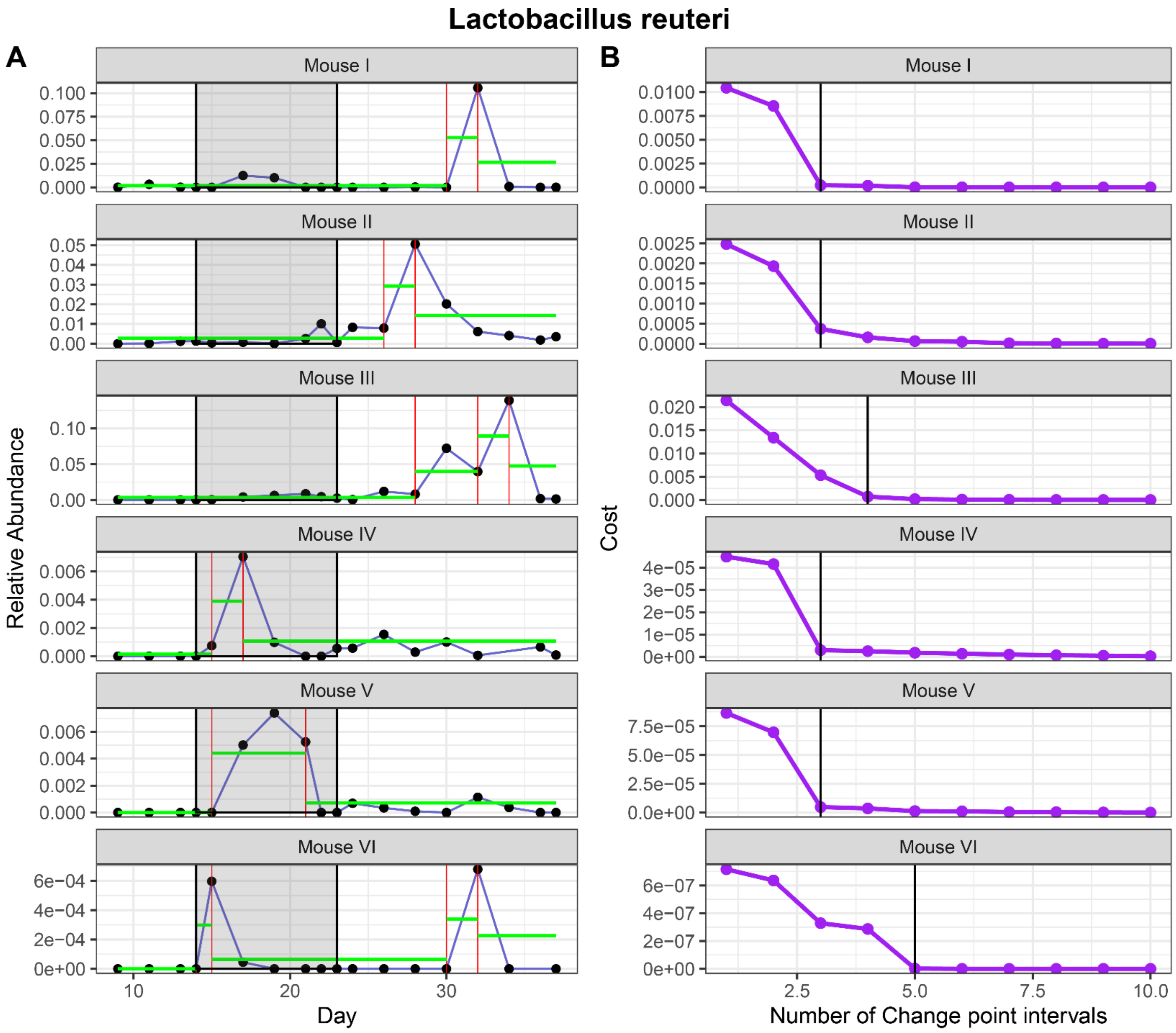

Figure S15: Change point analysis for *Lactobacillus reuteri* (A) and change point interval determination (B).

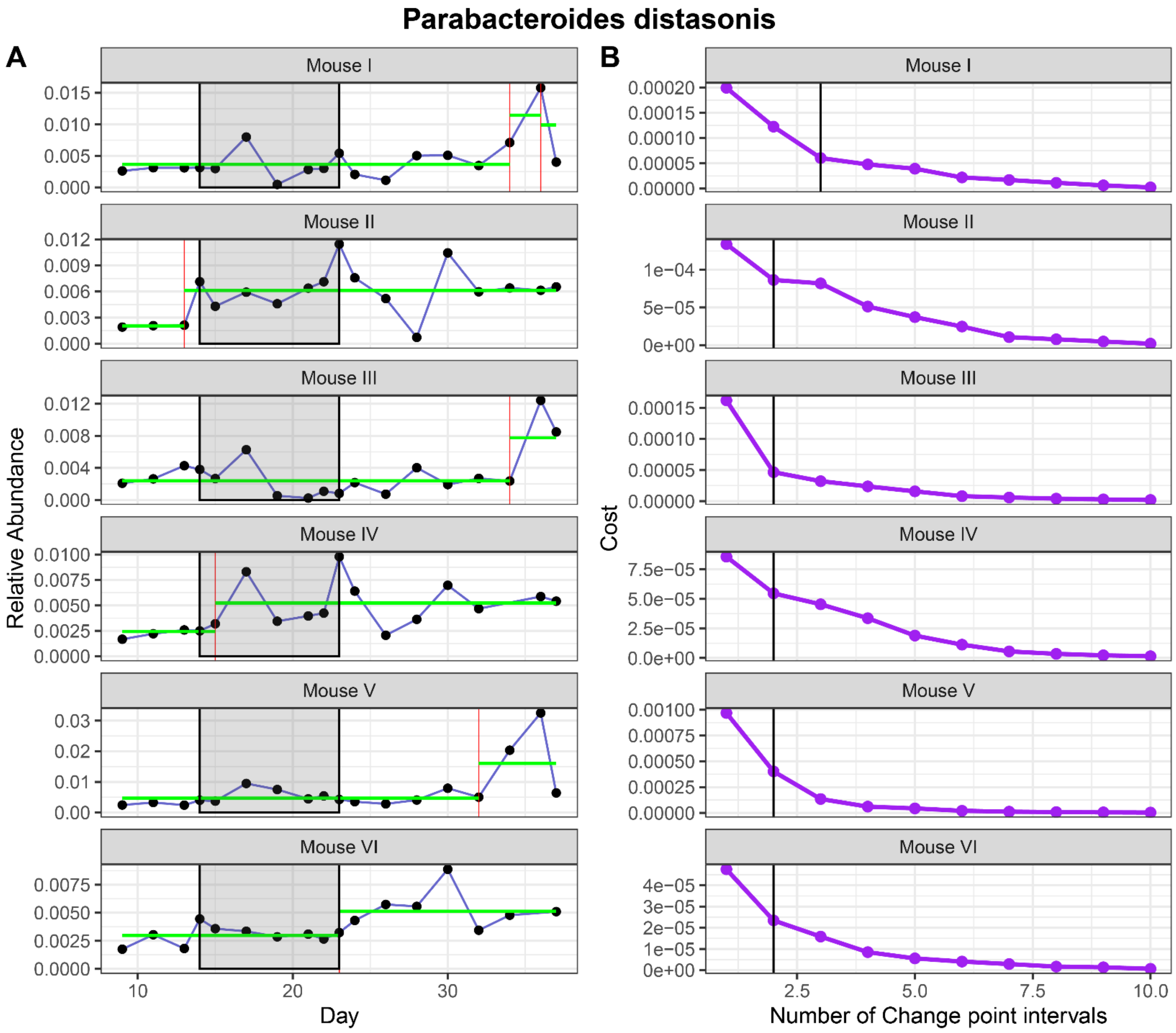

Figure S16: Change point analysis for *Parabacteroides distasonis* (A) and change point interval determination (B).

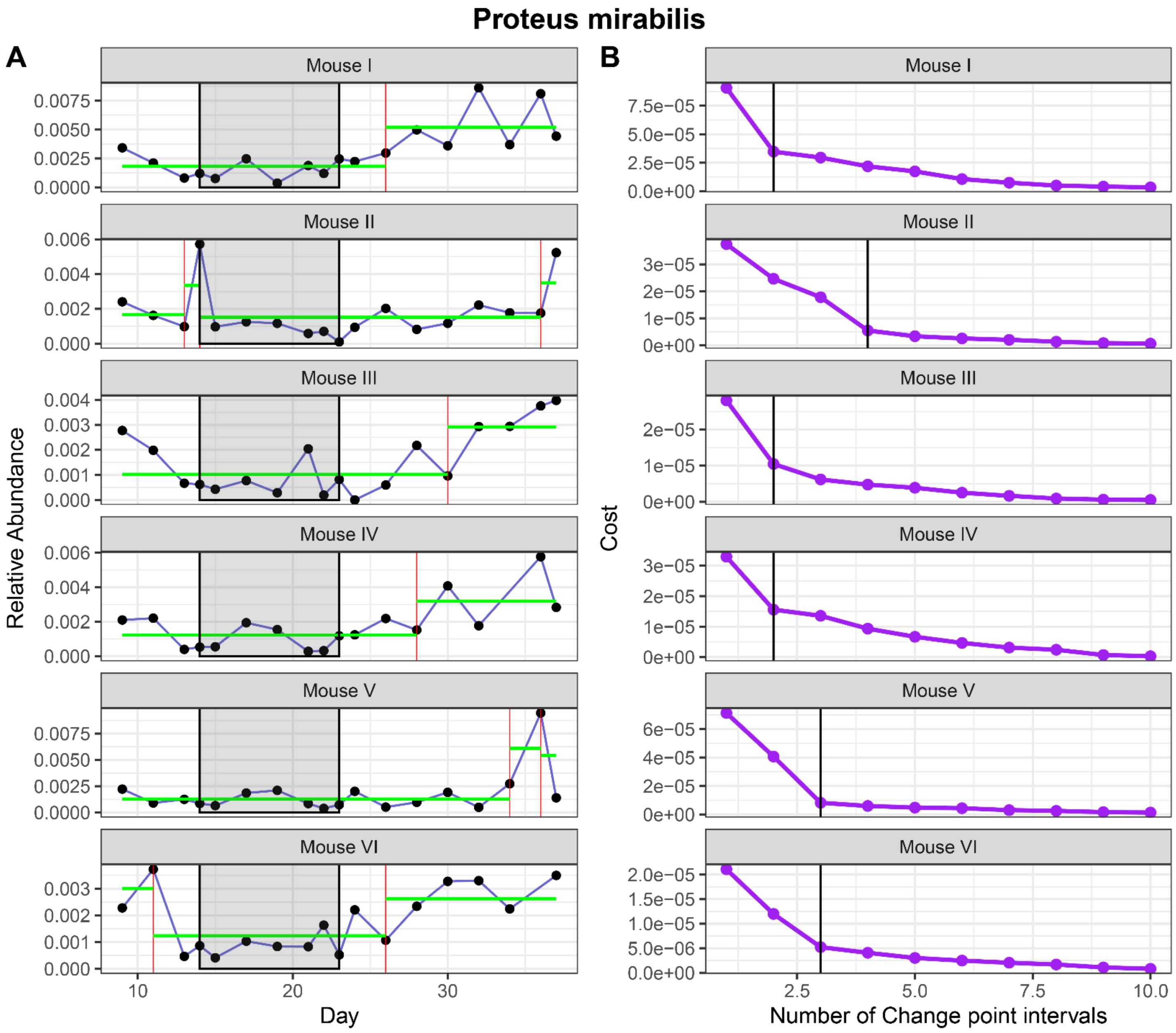

Figure S17: Change point analysis for *Proteus mirabilis* (A) and change point interval determination (B).
